# Digestive Aid as Adjunctive Therapy in Functional Dyspepsia: A Randomized Double-Blind Placebo-Controlled Clinical Trial

**DOI:** 10.7759/cureus.117706

**Published:** 2026-10-09

**Authors:** Raika Jamali, Amirhossein Aflatoonian, Abbas Keshtkar, Bardia Jamali, Yasaman Zarinfar, Eelia Gholampour Dehaki

**Affiliations:** 1 Research Development Center, Sina Hospital, Tehran University of Medical Sciences, Tehran, IRN; 2 Department of Internal Medicine, Sina Hospital, Tehran, IRN; 3 Department of Disaster &amp; Emergency Health, School of Public Health, Tehran University of Medical Sciences, Tehran, IRN

**Keywords:** clinica gastroenterology, digestive aid, dyspeptic symptoms, functional dyspepsia, herbal medicine

## Abstract

Functional dyspepsia (FD) is a common gastrointestinal complaint. This double-blind, placebo-controlled trial aims to determine the efficacy of a six-herb formulation (digestive aid) as a complementary therapy for FD. FD was confirmed based on Rome-IV criteria. Fifty patients were randomly assigned to receive either the digestive aid or a matching placebo for eight weeks, in addition to standard treatment protocols for FD. Dyspepsia symptoms were evaluated using the Leeds Dyspepsia Questionnaire (LDQ). The primary outcome was to compare changes in epigastric pain scores between the two groups from baseline to the end of treatment. The study included 30 (60%) female participants with a mean age of 40.8 (± 7.5) years. The comparison of mean changes in epigastric pain (-1.80 (-2.10, -1.50)), retrosternal pain (-2.12 (-2.40, -1.84)), regurgitation (-0.72 (-1.00, -0.44)), nausea (-1.40 (-1.70, -1.10)), early satiety (-0.88 (-1.16, -0.60)), belching (-1.00 (-1.30, -0.70)), dysphagia (-0.52 (-0.74, -0.30)), and vomiting (-0.40 (-0.64, -0.16)) showed significant improvement in the intervention group from baseline to the end of treatment. While a decrease in all dyspepsia domain scores was observed in the placebo group over the study period, the changes were not statistically significant. Between-group comparisons of mean differences revealed a significantly greater decrease in epigastric pain (-1.48 (-1.90, -1.06)), retrosternal pain (-1.96 (-2.40, -1.52)), regurgitation (-0.60 (-0.96, -0.24)), nausea (-1.32 (-1.72, -0.92)), early satiety (-0.76 (-1.14, -0.38)), belching (-0.84 (-1.24, -0.44)), and dysphagia (-0.40 (-0.68, -0.12)) in the digestive aid group compared to the placebo. The new formulation was well-tolerated with no major side effects during short-term use. Digestive aid, as an adjunct to standard therapy, may help alleviate symptoms in FD.

## Introduction

Functional dyspepsia (FD) is diagnosed by persistent dyspeptic symptoms in the absence of any organic gastrointestinal (GI) disorder [1]. It is a prevalent GI disorder, with earlier reports showing a high rate of up to 30% worldwide [2,3]. In Iran, the prevalence ranges from 2.2% to 29.9% [4]. FD imposes a substantial healthcare and economic burden. Pharmacological therapies often provide only modest symptom reduction and can be limited by side effects. The diversity of symptoms in FD forces patients to use multiple medications to alleviate their complaints [5]. This polypharmacy is inconvenient and may impact patient adherence.

To address the limitations in current treatments for FD, the use of supplementary herbal products has gained interest. Many medicinal plants possess carminative, pro-digestive, anti-inflammatory, or anxiolytic properties that may counter FD pathophysiology. Multi-herb preparations like the nine-ingredient formulation STW5 (Iberogast) have a long history of use in dyspepsia. A meta-analysis reported the superiority of Iberogast over placebo in decreasing dyspepsia symptom scores [6]. However, a recent systematic review rated the quality of evidence as low to moderate [7]. Additionally, a proprietary blend of peppermint oil and caraway oil (Menthacarin) has shown significant relief of FD symptoms [8]. Peppermint has antispasmodic effects on GI smooth muscle, and when combined with carminative agents like caraway or fennel, can alleviate bloating and pain [9]. Ginger (Zingiber officinale), with its prokinetic and antiemetic properties, has been shown to improve postprandial fullness, early satiety, and epigastric pain in uncontrolled trials [10]. Pimpinella anisum (anise), a traditional carminative, has produced remarkable reductions in dyspeptic symptoms in FD [11]. Gentian root (Gentiana spp.) has shown gastric pro-secretory and motility-stimulating effects in animal studies [12-14]. Fennel (Foeniculum vulgare) relaxes gut smooth muscle and alleviates gas. It was as effective as the anti-flatulent drug simethicone in reducing postoperative flatulence [9]. Althaea officinalis (marshmallow root) contains mucilage that may coat and soothe the gastric mucosa, reducing irritation and heartburn.

Combining these herbal agents into a unique supplement could be an attractive approach to target multiple symptoms simultaneously. Digestive aid is one such preparation that contains Althaea officinalis dry extract (marshmallow) (200 mg), Glycyrrhiza glabra dry extract (Liquorice) (50 mg), Zingiber officinale dry extract (ginger) (25 mg), Gentiana spp. dry extract (gentian) (125 mg), Foeniculum vulgare dry extract (fennel) (50 mg), Mentha piperita dry extract (peppermint) (50 mg), and Pimpinella anisum (anise) oil (20 mg). This supplement has been empirically used by physicians in Iran for dyspeptic symptoms, but no clinical trial has evaluated its efficacy. This trial tests a digestive aid added to omeprazole versus placebo added to omeprazole over eight weeks. The primary outcome is the between-group change in the epigastric pain score, and the secondary outcomes are the between-group change in other FD symptoms and adverse events.

## Materials and methods

Study design

This research was a randomized, double-blind, and placebo-controlled study conducted at a gastroenterology clinic in a tertiary hospital. The enrollment process started in April and ended in December 2025. The trial consisted of intervention (digestive aid) and placebo arms.

Ethical considerations

The current trial was carried out in line with the Declaration of Helsinki. The Research Ethics Committee of Sina Hospital approved the study protocol (Approval No. IR.TUMS.SINAHOSPITAL.REC.1404.010). The project was registered in ClinicalTrials.gov and IRCT (Registration numbers: NCT06958952 and IRCT20251202068196N1). All participants provided written informed consent after receiving a thorough explanation of the study aims and procedures. Participants were assured that they could withdraw at any time without consequence to their standard care. They were contacted every two weeks to encourage continued participation and address concerns. The herbal supplement and placebo were provided free of charge. The patients did not incur any additional costs during the study period.

Participant selection

Inclusion Criteria

Adults aged 18-75 with newly diagnosed FD were eligible. FD was defined by the Rome IV criteria [1]. All patients underwent a comprehensive evaluation by a gastroenterologist to document the diagnosis of FD. It included a detailed history, physical exam, laboratory tests, and upper GI endoscopy.

Exclusion Criteria

Those who received prior definitive treatment for dyspepsia, reported any known comorbid GI disease, were currently taking medications, had abnormal findings on exam or laboratory investigations, or had abnormal endoscopic findings, and those with known allergies to any components of the study supplement were excluded. Finally, pregnant or lactating women were not enrolled for safety considerations.

Randomization & blinding

We used block balanced randomization to enroll the participants into trial arms. Block sizes of four were utilized. A statistician performed the randomization using a computer-generated sequence. Allocation concealment was maintained using sequentially numbered, opaque sealed envelopes opened only at the time of assignment. Both active and placebo capsules were identical in appearance (opaque green film-coated pills). They were labeled with a code. The code was kept confidential by the pharmacist until all data collection was complete. Participants, clinicians, and outcome evaluators remained blinded to the assignment throughout the trial.

Interventions

The digestive aid capsules contained a proprietary blend of Althaea officinalis dry extract (marshmallow) (200 mg), Glycyrrhiza glabra dry extract (Liquorice) (50 mg), Zingiber officinale dry extract (ginger) (25 mg), Gentiana spp. dry extract (gentian) (125 mg), Foeniculum vulgare dry extract (fennel) (50 mg), Mentha piperita dry extract (peppermint) (50 mg), and Pimpinella anisum (anise) oil (20 mg). Participants assigned to the digestive aid arm took one capsule once daily, with water, after an evening meal. The placebo capsules were filled with inert microcrystalline cellulose.

Both digestive aid and the placebo were manufactured and quality-tested under GMP conditions by the sponsor company to ensure they were indistinguishable. The company had no role in study design, recruitment, data collection, statistical analysis, interpretation, manuscript preparation, or publication decision. We instructed patients in both groups to avoid initiating any new dyspepsia treatments (such as over-the-counter antacids, H2-blockers, or herbal remedies) during the study period. Rescue antacid (aluminum hydroxide/magnesium hydroxide chewable) was permitted for severe acute symptom flare-ups, but usage was tracked and kept minimal (and did not differ between groups). Each participant received a diary to record daily capsule intake and any adverse symptoms. An investigator who was blinded to the trial arms called the participants weekly to check on progress, encourage adherence, and discover adverse reactions.

It is worth mentioning that all participants in both treatment arms received omeprazole 20 mg once daily, initiated at the time of randomization, and continued throughout the eight-week intervention period. This background proton pump inhibitor (PPI) therapy was standardized to isolate the effect of the study supplement on dyspeptic symptoms.

Outcome measures

The Leeds Dyspepsia Questionnaire (LDQ) was used for assessing dyspepsia symptom severity. This is a valid and reliable instrument for quantifying dyspepsia symptoms [15]. The questionnaire was interviewer administered. It covers eight symptom domains: epigastric pain, retrosternal pain, regurgitation, nausea, vomiting, belching, early satiety, and dysphagia. Each item is scored by the patient on a Likert severity scale from 0 (no problem) to 5 (very severe). The primary outcome was comparing the mean change in epigastric pain score between the digestive aid and placebo. The secondary outcomes were defining the mean differences in retrosternal pain, regurgitation, nausea, vomiting, belching, early satiety, and dysphagia between the trial arms. The other outcomes were investigating the safety and possible side effects of the digestive aid.

Sample size calculation

Using a two-sided α=0.05 and power (1-β) of 0.90, we calculated that 46 patients in total would be required to detect a difference of that magnitude (effect size ~0.8) in a two-sample comparison [16]. To account for potential dropouts or loss to follow-up, we planned to recruit 50 patients (25 in each arm).

Statistical analysis plan

We assessed baseline comparability of the two groups on demographic and clinical variables using t-tests for continuous variables and chi-square tests for categorical variables. Continuous outcome measures were summarized as means (± standard deviations).

Within-group changes were summarized descriptively with mean changes and 95% confidence intervals; treatment efficacy (LDQ score change) was assessed primarily by adjusted between-group comparisons using analysis of covariance (ANCOVA).

The primary analysis used ANCOVA with end-of-treatment epigastric-pain score as the dependent variable, treatment group as the independent variable, and baseline epigastric-pain score as a covariate. We set the significance level at α = 0.05 (two-tailed) for the primary analysis. All statistical analyses were carried out using SPSS software, Version 17 (Released 2008; SPSS Inc., Chicago, IL).

## Results

Participants’ flowchart and characteristics

A total of 58 individuals were assessed for eligibility. Eight individuals were excluded before randomization due to ineligibility or declining participation. Finally, 50 met all eligibility criteria and were randomized to either the digestive aid (n=25) or placebo (n=25) arm. All randomized participants received their allocated intervention, completed the eight-week follow-up, and were included in the final analysis. The high level of adherence is likely supported by the brief trial duration and regular follow-up contacts, which contributed to excellent compliance and no attrition. The flowchart diagram is shown in Figure 1.

**Figure 1:**
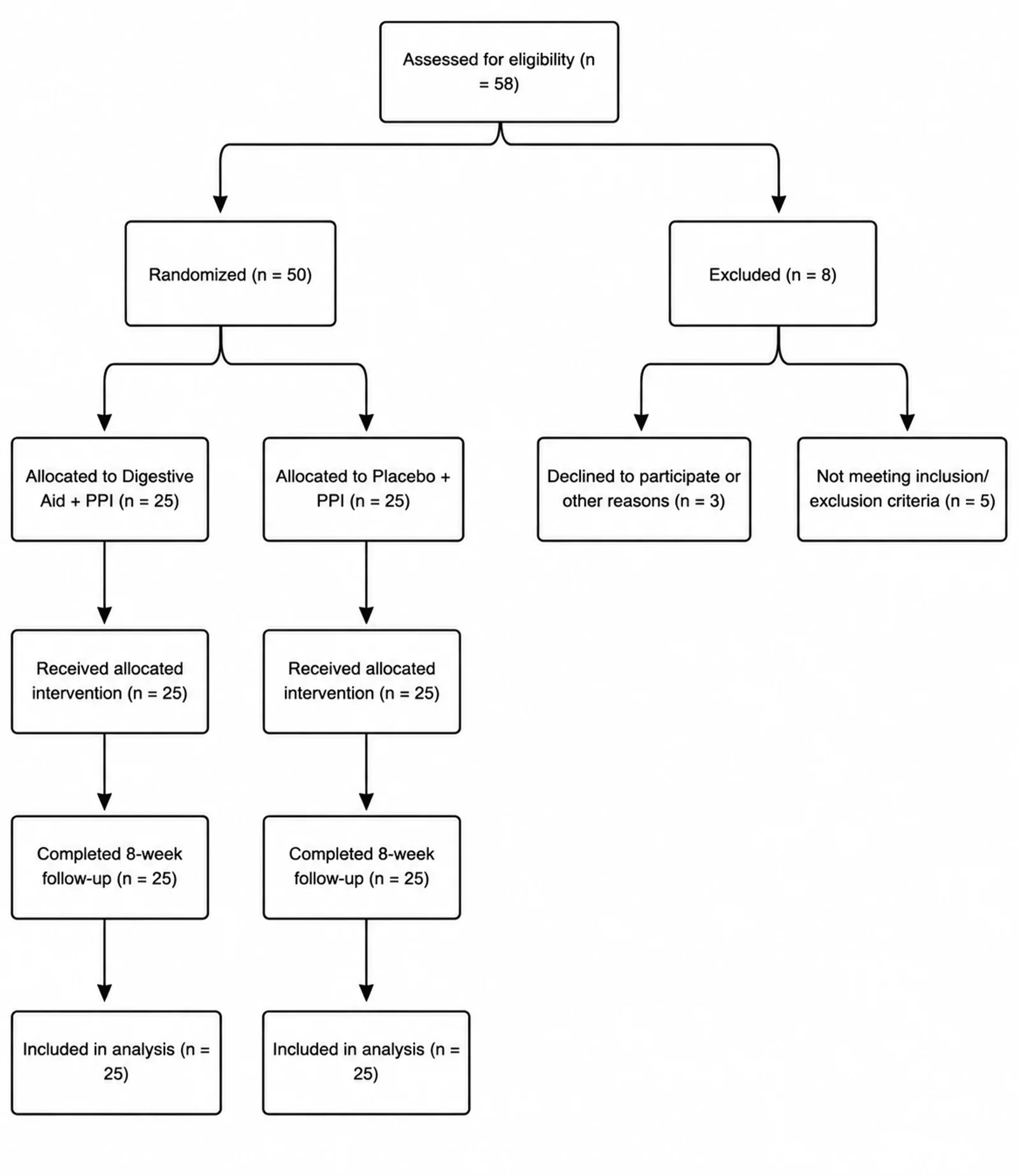
CONSORT flow diagram showing participant progression through the study. CONSORT: Consolidated Standards of Reporting Trials; PPI: proton pump inhibitor

This project consisted of 30 female participants (60%). The randomized cohort had a mean age of 40.8 (± 7.5) and ranged between 22 and 56 years. There were no statistically significant differences between the study arms in age, gender, body mass index, and dyspepsia symptom scores at baseline (Table 1).

**Table 1:** The baseline demographic and clinical characteristics of the study participants. Data are presented as mean (standard deviation) or n (%). The p-value indicates the comparisons of two study groups at baseline. T test (*) is used for continuous variables and chi-square test (**) for categorical variables.

Characteristics	Placebo (n = 25)	Digestive Aid (n = 25)	P-value
Age (years)	41.0 (10.6)	40.7 (6.0)	0.91 *
Sex			
Male	10 (40%)	11 (44%)	0.78 **
Female	15 (60%)	14 (56%)	
Body mass index (kg/m²)	30.9 (3.7)	30.7 (3.2)	0.83 *
Symptom score			
Epigastric pain	4.12 (0.67)	4.00 (0.91)	0.60 *
Retrosternal pain	3.96 (0.79)	4.08 (0.76)	0.59 *
Regurgitation	3.40 (0.71)	3.28 (0.68)	0.54 *
Nausea	3.60 (0.91)	3.44 (0.82)	0.52 *
Vomiting	1.76 (0.78)	1.72 (0.74)	0.85 *
Belching	2.84 (0.94)	2.96 (0.84)	0.64 *
Early satiety	2.80 (0.65)	2.64 (0.76)	0.43 *
Dysphagia	1.84 (0.62)	1.92 (0.70)	0.67 *

Within-group analysis

The dyspepsia symptom scores and their changes during the study period according to the trial arms are provided in Table 2.

**Table 2:** The change in dyspepsia symptom scores according to trial arms during the study period. Data are presented as mean (standard deviation). The p-value indicates the comparison of the mean dyspepsia symptom score from baseline to the end of treatment in study groups (within-group analysis by ANCOVA). ANCOVA: Analysis of covariance

Symptom	Group	Baseline Mean (SD)	End-of-Treatment Mean (SD)	Within-Group Change Mean (95% CI)	P-value (Within-Group)
Epigastric pain	Placebo	4.12 (0.67)	3.80 (0.91)	−0.32 (−0.70, 0.06)	0.09
	Digestive Aid	4.00 (0.91)	2.20 (0.76)	−1.80 (−2.10, −1.50)	<0.001
Retrosternal pain	Placebo	3.96 (0.79)	3.80 (0.91)	−0.16 (−0.60, 0.28)	0.46
	Digestive Aid	4.08 (0.76)	1.96 (0.89)	−2.12 (−2.40, −1.84)	<0.001
Regurgitation	Placebo	3.40 (0.71)	3.28 (0.74)	−0.12 (−0.40, 0.16)	0.39
	Digestive Aid	3.28 (0.68)	2.56 (1.04)	−0.72 (−1.00, −0.44)	<0.001
Nausea	Placebo	3.60 (0.91)	3.52 (0.87)	−0.08 (−0.40, 0.24)	0.6
	Digestive Aid	3.44 (0.82)	2.04 (0.89)	−1.40(−1.70, −1.10)	<0.001
Early satiety	Placebo	2.80 (0.65)	2.68 (0.99)	−0.12 (−0.50, 0.26)	0.52
	Digestive Aid	2.64 (0.76)	1.76 (0.60)	−0.88 (−1.16, −0.60)	<0.001
Belching	Placebo	2.84 (0.94)	2.68 (1.03)	−0.16 (−0.52, 0.20)	0.37
	Digestive Aid	2.96 (0.84)	1.96 (0.79)	−1.00 (−1.30, −0.70)	<0.001
Dysphagia	Placebo	1.84 (0.62)	1.72 (0.68)	−0.12 (−0.30, 0.06)	0.18
	Digestive Aid	1.92 (0.70)	1.40 (0.50)	−0.52 (−0.74, −0.30)	<0.001
Vomiting	Placebo	1.76 (0.78)	1.68 (0.75)	−0.08 (−0.28, 0.12)	0.42
	Digestive Aid	1.72 (0.74)	1.32 (0.48)	−0.40 (−0.64, −0.16)	0.003

The within-group analysis revealed that digestive aid supplement therapy produced significant improvements across all dyspepsia domain scores. The mean scores for belching (Figure 2A), early satiety (Figure 2B), nausea (Figure 2C), retrosternal pain (Figure 2D), dysphagia (Figure 2E), epigastric pain (Figure 2F), regurgitation (Figure 2G) and vomiting (Figure 2H) were significantly decreased during the study period in the digestive aid arm. While the dyspepsia domain scores also decreased in the placebo arm, the reduction was not statistically significant (Figure 2). It is worth mentioning that a trend in epigastric pain (Figure 2F) improvement was seen while taking the placebo (P: 0.09).

**Figure 2:**
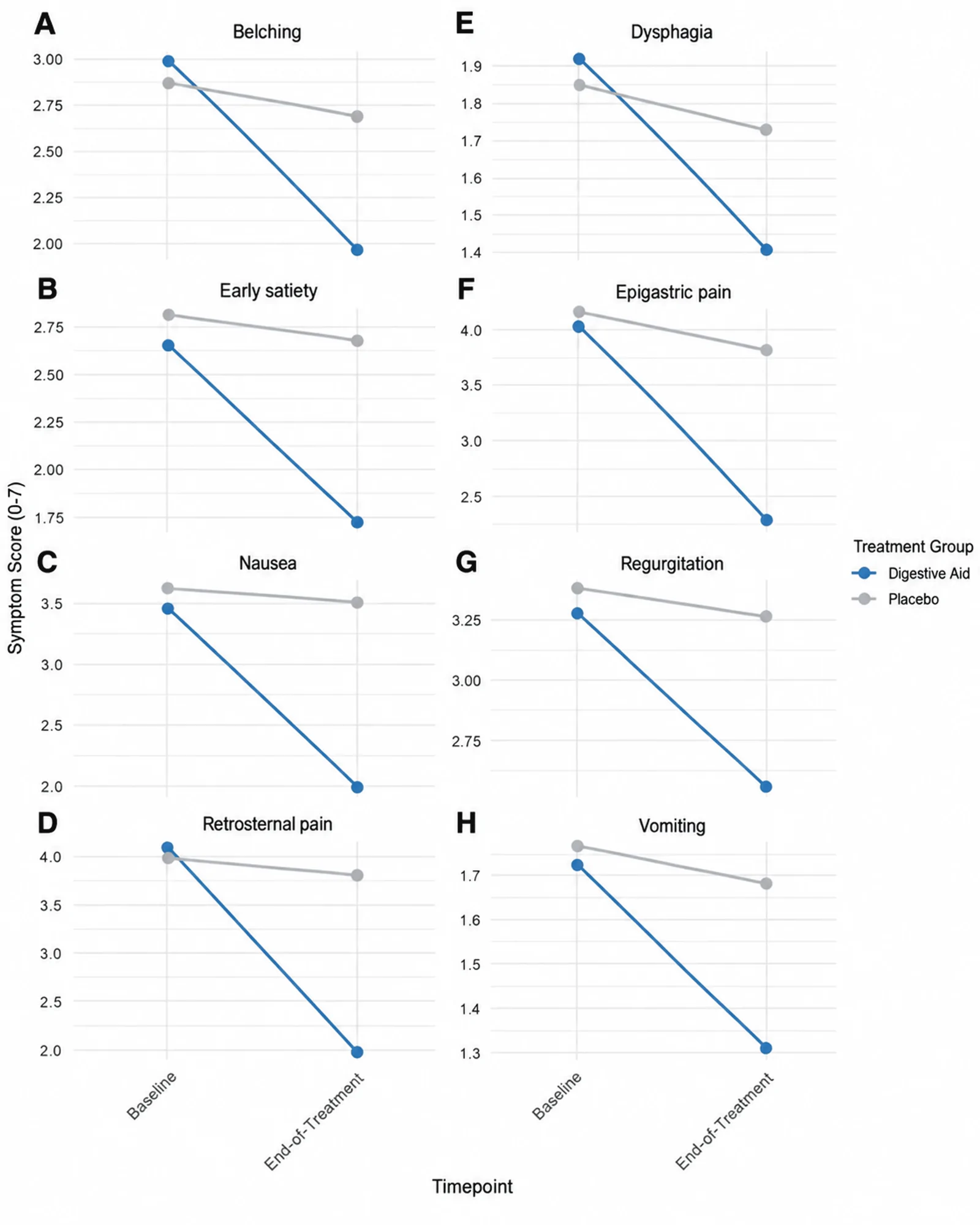
The changes in dyspepsia symptom scores within the trial arms during the study period. Changes in mean dyspepsia symptom scores from baseline to the end of treatment in the digestive aid and placebo groups. Panels represent (A) belching, (B) early satiety, (C) nausea, (D) retrosternal pain, (E) dysphagia, (F) epigastric pain, (G) regurgitation, and (H) vomiting. Within-group changes were analyzed using ANCOVA. ANCOVA: Analysis of covariance

Between-group analysis

The comparisons of between-group mean dyspeptic symptom scores changes during the study period are demonstrated in Figure 3. The between-group mean differences analysis revealed a significantly greater decrement in epigastric pain (−1.48 (−1.90, −1.06)), retrosternal pain (−1.96 (−2.40, −1.52)), regurgitation (−0.60 (−0.96, −0.24)), nausea (−1.32 (−1.72, −0.92)), early satiety (−0.76 (−1.14, −0.38)), belching (−0.84 (−1.24, −0.44)), and dysphagia (−0.40 (−0.68, −0.12)) in the digestive aid group than in the placebo group (P values: <0.001, <0.001, 0.001, < 0.001, < 0.001, <0.001 and 0.007 respectively). Meanwhile, there was a trend toward a mean difference reduction in vomiting (−0.32 (−0.68, 0.04)) in the intervention group when compared with the placebo group (P value: 0.08).

**Figure 3:**
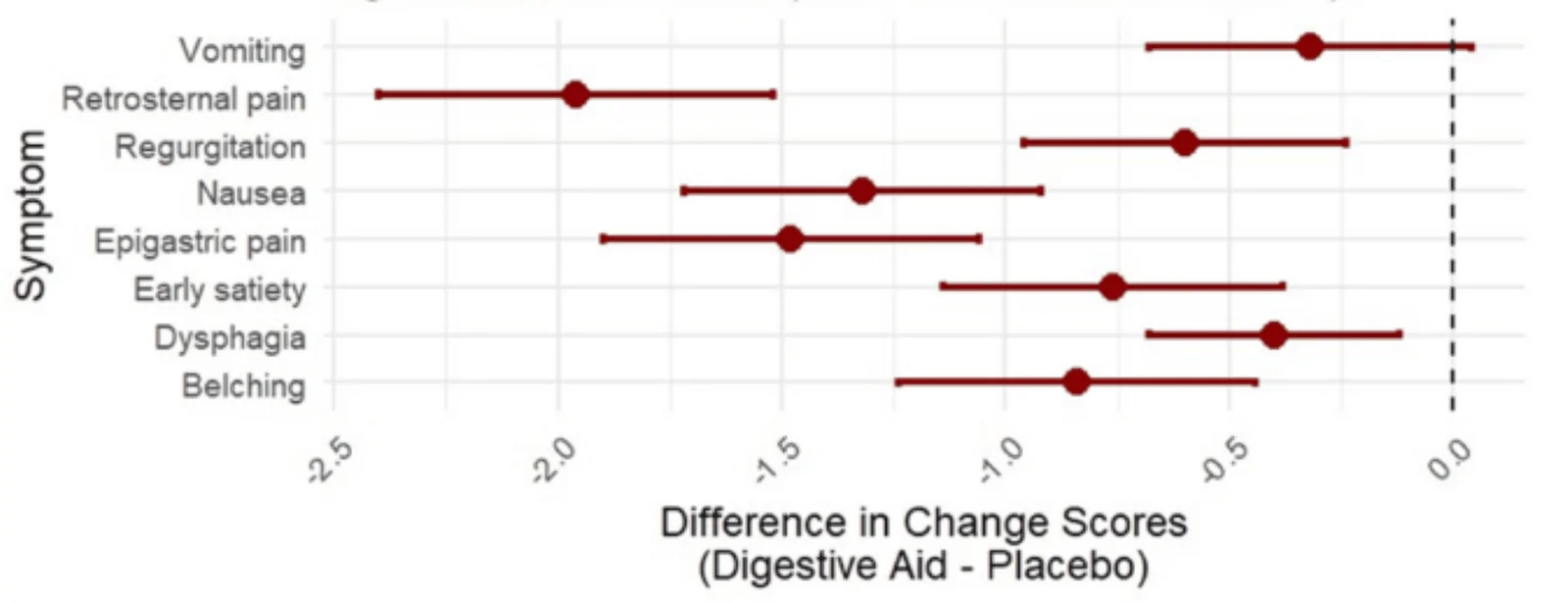
The comparison of mean differences changes in dyspepsia symptom scores between digestive aid and placebo. Negative values indicate greater improvement with digestive aid (between-group analysis by ANCOVA). ANCOVA: Analysis of covariance

Safety and tolerability

Digestive aid was well tolerated, and no serious adverse events were recorded. Only minor, transient adverse effects were reported. Three patients in the digestive aid group (12%) reported mild bloating or gas during the first week of therapy, which resolved spontaneously; two patients in the placebo group (8%) reported similar transient bloating. One patient receiving digestive aid noted an occasional mild peppermint aftertaste or peppermint-scented belching, which was not considered bothersome and did not lead to unblinding during the trial. Treatment adherence was high in both groups. Mean pill-count adherence was 95% (±3) in the digestive aid group and 94% (±2) in the placebo group; no participant had adherence below 92%.

## Discussion

While reviewing the latest literature about FD, no published data regarding the efficacy of "digestive aid" in Iran were found. This herbal cocktail, when used adjunctively to standard therapy, resulted in significantly greater improvement in epigastric pain, retrosternal pain, regurgitation, nausea, belching, early satiety, and dysphagia compared to patients who only received standard therapy. Importantly, digestive aid was well tolerated and did not cause more adverse effects than the placebo.

In this small, single-center trial, digestive aid plus omeprazole was associated with greater short-term improvement in several patient-reported FD symptoms than omeprazole plus placebo. The between-group mean differences analysis revealed a significantly greater decrement in epigastric pain (−1.48 (−1.90, −1.06)), retrosternal pain (−1.96 (−2.40, −1.52)), regurgitation (−0.60 (−0.96, −0.24)), nausea (−1.32 (−1.72, −0.92)), early satiety (−0.76 (−1.14, −0.38)), belching (−0.84 (−1.24, −0.44)), and dysphagia (−0.40 (−0.68, −0.12)) in the digestive aid group than in the placebo group.

FD is a common disorder of the gut-brain axis with a chronic relapsing course and a significant impact on quality of life. Current guidelines suggest small gains with PPIs, prokinetics, and psychotropic drugs, emphasizing the need for safer, multi-mechanistic treatments. Complementary medicine is used in patients with abdominal pain [17], and our trial adds to the evidence that carefully designed herbal combinations may help patients with FD.

Our findings are consistent with existing research indicating that specific herbal formulations can enhance outcomes for FD. A comprehensive systematic review and meta-analysis of herbal treatments for FD found that herbal preparations significantly improved global dyspepsia symptoms compared to placebo. However, concerns were raised about the heterogeneity of the studied populations and the risk of bias [18]. A Cochrane review in 2023 focused on non-Chinese herbal medicines in 41 RCTs including 4477 participants. It concluded that STW5 (Iberogast), curcumin, and peppermint/caraway combinations might improve global FD symptoms compared to placebo. The results had low to very low certainty due to small sample sizes, methodological limitations, and limited head-to-head data against standard drugs [19]. Narrative reviews have also reached similar conclusions, suggesting that herbal medicines show promise and are often well tolerated. However, the evidence base has been dominated by a few products, such as STW5 and peppermint/caraway oil, and short-term studies until recently [20]. Our trial contributes to this literature by providing randomized, double-blind, placebo-controlled data for a distinct six-herb preparation that combines demulcent, prokinetic, bitter, and carminative components in a single capsule. Multi-component formulations such as STW5 (Iberogast) and Menthacarin (peppermint plus caraway oil) are particularly relevant comparators. A meta-analysis of double-blind RCTs of STW5 showed an overall therapeutic effect in FD with good tolerability, and recent work with an updated formulation (STW5-II) confirms improvements in fullness, early satiety, and global symptom scores compared to placebo [6,7,21,22]. Similarly, several RCTs and pooled analyses of peppermint/caraway combinations (Menthacarin) have shown significant short-term relief of FD symptoms compared to placebo, with effects on both postprandial distress and epigastric pain syndromes [8,23,24]. The response pattern seen with digestive aid resulted in broad improvement in both PDS and EPS symptoms, rather than selective benefit on a single symptom cluster, aligning with the emerging paradigm of multi-target herbal interventions for FD. Digestive aid contains Althaea officinalis (marshmallow) root, Zingiber officinale (ginger), Gentiana spp. (gentian), Foeniculum vulgare (fennel), Mentha Piperita (peppermint), and Pimpinella Anisum (anise) oil, each with mechanistic actions relevant to FD. Ginger has been shown to accelerate gastric emptying and improve upper GI symptoms in FD [10]. Gentian is a classic bitter agent that enhances gastric secretion and motility, likely via bitter-taste receptors and downstream effects on gastric functions [14,25,26]. Fennel and anise provide carminative and antispasmodic effects that can reduce gas, bloating, and postprandial cramping in FD [11,27]. Peppermint oil has demonstrated efficacy in reducing visceral pain and dyspeptic symptoms through calcium channel blocking and smooth muscle relaxing properties [23-25]. Marshmallow root provides demulcent mucilage that can coat and protect the upper gastrointestinal mucosa, supported by preclinical data suggesting gastroprotective and anti-inflammatory effects [28]. The digestive aid formulation combines agents that may act on gastric accommodation, mucosal protection, motility, gas handling, and visceral hypersensitivity. While our trial was not designed to delineate mechanisms, the symptom improvement aligns with other complex herbal preparations studied in FD [20].

FD trials are notoriously characterized by high placebo response rates. Recent meta-analyses of pharmacologic FD trials report pooled placebo response rates of 39 to 44%, depending on the responder definition and study design [29]. In our study, the placebo group showed only modest change in the primary symptom scale, and very few subjects achieved near-remission thresholds, despite similar baseline severity. The modest placebo response and observed between-group differences are compatible with a treatment effect, but confirmation in larger trials is required. At the same time, the reliance on patient-reported outcomes underscores the importance of including objective or mechanistic endpoints (e.g., gastric emptying, accommodation, or mucosal markers) in future confirmatory studies [30].

Taken together with the wider literature, our findings suggest that digestive aid could be considered as a candidate adjunct or alternative therapy in FD. This is particularly true for patients with prominent postprandial fullness, early satiety, and epigastric pain who have inadequate relief with standard approaches or prefer herbal treatments. The multi-symptom improvement observed parallels that reported with STW5 and peppermint/caraway combinations. However, considering the chronic relapsing nature of FD, the frequent need for long-term symptom management is warranted [6-8,23,25]. Nevertheless, current international guidelines do not yet position herbal products as first-line therapies. This is largely because of heterogeneous evidence, regulatory variability, concerns about quality control, and rare adverse events [20]. We suggest that digestive aids may be best used within a stepped-care model. After appropriate management, including H. pylori testing and lifestyle and dietary modification, it could be offered as an adjunct to standard therapy in motivated patients. Close monitoring for benefit and any adverse effects is recommended. Future trials should examine whether use of digestive aid reduces the need for additional symptom-directed medicines.

Key limitations include the single-center design, small sample, short treatment duration, reliance on patient-reported outcomes, limited assessment of psychiatric comorbidity, and absence of long-term follow-up.

This trial opens several avenues for future research. One is to conduct long-term studies to see if continuous use of digestive aid maintains symptom control and whether it can prevent FD relapse. Another direction is substituting the subjective outcomes (symptom based) with the objective ones (pathophysiologic based). For instance, measuring gastric emptying (by scintigraphy) and gastric accommodation (by barostat or MRI) in patients on digestive aid versus placebo would help delineate how it exerts effects. Additionally, assessing gastric mucosal biopsies for inflammatory cell counts before and after therapy could test the supplement’s anti-inflammatory effects. Furthermore, exploring each ingredient’s contribution (perhaps via factorial trial or removing one component at a time) could identify if a simplified formula might suffice or if the full combination is necessary for maximal effect. Finally, given the positive findings regarding the application of digestive aid, a larger multicenter trial is recommended to evaluate efficacy, safety, and other important endpoints like the quality of life and cost-effectiveness.

## Conclusions

In this eight-week, single-center trial, digestive aid plus omeprazole was associated with improved patient-reported dyspepsia symptoms and no serious adverse events in patients with FD. Over eight weeks, digestive aid led to substantial reductions in epigastric pain, postprandial fullness, early satiety, and nausea compared to placebo. The broad symptom relief and well tolerability observed suggest that this herbal combination may address multiple pathophysiological facets of FD, making it a promising candidate for further investigation as a complementary treatment. No safety concerns emerged during the trial, and patient-reported symptom relief indicated meaningful gains in the treatment group. These preliminary findings support the potential utility of digestive aid as an adjunctive therapy for FD, particularly in patients seeking non-pharmacologic options. However, its use in clinical practice should await confirmation through larger, multicenter, and longer-term trials.
